# *Phytopythium helicoides* Associated with Root Rot of Sweet Cherry in Chile: Identification, Pathogenicity, and Incidence of Decline Symptoms

**DOI:** 10.3390/plants15192926

**Published:** 2026-09-24

**Authors:** Diyanira Castillo-Novales, Natalia Riquelme, Claudio Henríquez, Catalina Ferreira, Pia Varas, Ximena Besoain

**Affiliations:** 1 Laboratorio de Fitopatología, Escuela de Agronomía, Pontificia Universidad Católica de Valparaíso, San Francisco s/n La Palma, Quillota 2260000, Chile; diyaniracastillonovales@gmail.com (D.C.-N.); natalia.riquelme@pucv.cl (N.R.); claudio.henriquez@gmail.com (C.H.); catalina.ferreira.fs@gmail.com (C.F.); pia.varas@pucv.cl (P.V.); 2 Millennium Nucleus Bioproducts, Genomics and Environmental Microbiology (BioGEM), Avenida España 1680, Valparaíso 2390123, Chile

**Keywords:** *Phytopythium helicoides*, Prunus avium, root rot, oomycete, Lapins cultivar, MaxMa14 rootstock, ITS, COX I, β-tubulin

## Abstract

Species within the genus *Phytopythium* have recently been recognized as significant soilborne pathogens impacting various crops globally. However, their occurrence and pathogenicity in sweet cherry (*Prunus avium*) have not been studied in Chile before. This study aimed to identify *Phytopythium* isolates associated with root rot decline in cherry plants and to assess their pathogenicity under controlled conditions and in the field. Symptoms were recorded, and cherry plants grafted onto Maxma14 samples were collected from a commercial orchard in the Valparaíso region. We obtained isolates using selective media and characterized them based on morphological characteristics and molecular analysis of the internal transcribed spacer (ITS), cytochrome c oxidase subunit I (COX I), and β-tubulin regions. Phylogenetic analysis based on the three concatenated regions supported the placement of the three isolates within the *Phytopythium. helicoides* clade. Incidence across three sectors of a commercial orchard ranged from 24.3% to 42.8%. Pathogenicity tests involved inoculating one-year-old *Prunus avium* ‘Lapins’ plants grafted onto MaxMa14 rootstock by drenching the substrate with 300 mL of a zoospore suspension (1 × 10^4^ zoospores/mL) and evaluating them 90 days post-inoculation. Inoculated plants exhibited reduced growth, foliar symptoms, and root necrosis, compared with non-inoculated controls. These findings confirm that *Phytopythium helicoides* can be pathogenic in cherry trees and underscore its emerging role as a soilborne pathogen in Chilean cherry production systems.

## 1. Introduction

Soilborne oomycetes are important causes of root and crown rot in agricultural crops, including fruit trees, vegetables, and ornamental plants. *Phytophthora* species have long been recognized as major pathogens in fruit production, while *Pythium* species are also associated with root diseases in a broad range of agricultural hosts [1,2,3]. Within this group, the genus *Phytopythium* (*Phy.*) has gained increasing attention because several species are associated with root rot and plant decline in horticultural and woody crops [4,5]. Many of these organisms were formerly classified within *Pythium* clade K. Phylogenetic analyses based on nuclear large-subunit (LSU) and small-subunit (SSU) rDNA sequences, together with mitochondrial cytochrome c oxidase subunit I (cox1), supported the recognition of *Phy.* as a distinct genus [4]. Additional molecular markers, including β-tubulin, have also been used to investigate species identity and relationships within the genus [6]. 

Historical treatments of *Pythium* compiled species descriptions and illustrations that provided a morphological basis for taxonomic comparisons before the modern delimitation of *Phy.* [7]. Members of *Phy.* exhibit a combination of morphological characteristics found in *Pythium* and *Phytophthora*. These include papillate sporangia with internal proliferation and zoospore differentiation within an external vesicle [4]. Although these features are useful for identification, their expression under culture conditions and their similarity to structures produced by related oomycetes can complicate routine diagnosis. Integrating morphological observations with molecular analyses is therefore important for reliable identification [4,8]. Accurate identification also provides a basis for interpreting pathogenicity results and distinguishing the contributions of different oomycetes to root disease complexes [9].

Reports from different crops and geographic regions illustrate the phytopathological relevance of *Phy*. For example, *Phytopythium helicoides* has been reported to cause crown rot of strawberry and crown and root rot of *Rhododendron pulchrum*, while *Phytopythium chamaehyphon* has been associated with kiwifruit vine decline [10,11,12]. Studies of kiwifruit decline have also examined the involvement of multiple *Phy.* species, emphasizing the need to identify the organisms present and evaluate their pathogenicity rather than infer their role from disease symptoms alone [13]. In Japan, *Phy. helicoides* and *Phy. vexans* were identified among five oomycete species causing root and stem rot of sedum, with *Phy. helicoides* showing high virulence in pathogenicity tests [14]. In Brazil, *Phy.* sp. was reported to cause storage root rot of cassava (Manihot esculenta), with pathogenicity confirmed by experimental inoculation and re-isolation from symptomatic root tissues [15]. Together, these reports demonstrate that members of the genus can affect both herbaceous and woody hosts and should be considered in investigations of root rot and plant decline.

Water availability is an important component of the biology and epidemiology of soilborne oomycetes. Under favorable moisture conditions, zoospore production and movement facilitate contact with susceptible plant tissues, while irrigation water can provide a pathway for pathogen dissemination [8,16,17]. Species of *Phy.*, together with *Pythium* and *Phytophthora*, have been detected in recycled irrigation water in container nurseries [18]. Their occurrence in these systems matters for plant health because nursery production brings together susceptible planting material, irrigation water, and substrates that may support pathogen survival and infection. Nevertheless, detecting an organism in water or plant-associated samples does not by itself establish its contribution to disease, making experimental pathogenicity assessment essential.

In woody perennial crops, root diseases can compromise plant establishment and subsequent development. Within *Prunus*, rootstock selection matters because responses to soilborne pathogens vary among host materials and pathogen species [17,19]. *Phytopythium vexans* has been reported to cause root and crown rot of flowering cherry (*Prunus serrulata*) in Tennessee [20]. This report establishes pathogenicity on an ornamental cherry species, but it should be distinguished from evidence obtained on sweet cherry (*Prunus avium*). Beluzán et al. [19] evaluated several oomycete species on the *Prunus* hybrid rootstocks ‘Garnem’, ‘GF-677’, and ‘Rootpac-40’ and demonstrated the pathogenic potential of *Phy. helicoides* in these host materials. These findings provide relevant context for investigating *Phy.* in stone fruit production, although results from these hybrid rootstocks cannot be directly extrapolated to sweet cherry plants grafted onto MaxMa14.

Sweet cherry is an economically important fruit crop in Chile, with a substantial role in the country’s fresh fruit export sector [21]. The establishment of productive orchards depends in part on healthy planting material and adequate root development. Root deterioration and decline in young trees therefore warrant investigation, particularly when symptoms may involve soilborne pathogens. However, reduced vigor, leaf chlorosis, wilting, and root deterioration are not specific to a single causal agent. Field assessments based on these symptoms describe the extent of plant decline but require complementary diagnostic evidence before attributing the observed incidence to a particular pathogen.

Despite reports of *Phy.* diseases in other crops and *Prunus* hosts, its association with root rot and its pathogenicity in sweet cherry in Chile remain poorly documented. Addressing this knowledge gap requires integrating field observations, isolation from symptomatic tissues, morphological and molecular identification, and inoculation assays. This approach can determine whether recovered isolates can cause disease while distinguishing experimentally demonstrated pathogenicity from the causes of decline across an entire orchard.

Observations of root deterioration and decline symptoms in a two-year-old commercial sweet cherry orchard in the Valparaíso region prompted the present investigation. This study aimed to characterize *Phytopythium* isolates recovered from symptomatic roots using morphological observations and multilocus molecular and phylogenetic analyses, quantify the incidence of decline symptoms across three orchard sectors, and evaluate the pathogenicity of the isolates on ‘Lapins’ sweet cherry plants grafted onto MaxMa14 rootstock in two independent inoculation assays.

## 2. Results

### 2.1. Morphological Characterization of Isolates

Isolates PUCV 2372 and PUCV 2368 were morphologically characterized; the results are presented in Figure 1A,B. Both isolates were identified as *Phy. helicoides*, exhibited similar colony morphology, with white, aerial mycelium and no specific growth pattern on corn meal agar (CMA) plates. The average radial growth rate across both isolates was 15.75 mm day^−1^, corresponding to mean colony diameters of 66 ± 2 mm for PUCV 2372 and 70 ± 3 mm for PUCV 2368 after 48 h.

Isolate PUCV 2372 mainly produced ellipsoid, papillate sporangia (23.4 ± 2.3 × 14.6 ± 1.4 µm, *n* = 30), with a few globose, non-papillate sporangia also present. Sporangia frequently formed discharge tubes, and internal proliferation was observed. Isolate PUCV 2368 mainly produced globose, non-papillate sporangia (19.3 ± 2.9 µm in diameter, *n* = 30), with occasional papillate and ovoid sporangia (Figure 1C–F).

Sexual structures were not observed in either isolate under sporangium-producing conditions during the study period (two weeks). In both isolates, the hyphae were smooth and without hyphal swelling (Figure 1C–F).

### 2.2. Molecular Identification of the Isolates and Phylogenetic Analysis

BLASTn (BLAST+ version 2.17.0) analysis showed high sequence identity between the three Chilean isolates and reference sequences deposited in GenBank (Table 1). For isolates PUCV 2369 and PUCV 2372, ITS, β-tubulin, and COX I showed the highest sequence identity with *Phy. helicoides*, with identity values ranging from 99.13% to 99.71%. In contrast, isolate PUCV 2368 showed discordant results among markers. Its ITS sequence showed 100% identity with *Phy. chamaehyphon* (GenBank accession PP087944.1), whereas its β-tubulin and COX I sequences showed the highest identity with *Phy. helicoides*, with 99.65% and 100% sequence identity, respectively.

Phylogenetic analysis of the concatenated ITS, β-tubulin, and COX I dataset placed the three Chilean isolates within the *Phy. helicoides* clade (Figure 2). This clade was separated from *Phy. chamaehyphon* with high bootstrap support (99%). Isolate PUCV 2372 clustered with the *Phy. helicoides* H5 reference isolate with 94% bootstrap support. Despite the 100% ITS sequence identity of PUCV 2368 with *Phy. chamaehyphon* in the BLASTn analysis, the concatenated multilocus analysis placed PUCV 2368 within the *Phy. helicoides* clade.

### 2.3. Incidence of Decline Symptoms in the Orchard

Field evaluation revealed a considerable proportion of affected plants across the three orchard sectors evaluated. Disease incidence, defined as the proportion of plants classified as mildly affected or medium to severely affected, ranged from 24.3% to 42.8% across the assessed sectors. Sector 2 recorded the highest incidence (42.8%; 978/2.285), followed by Sector 3 (32.5%; 695/2.136) and Sector 1 (24.3%; 306/1.260) (Table 2). These values represent the incidence of visually assessed decline symptoms on cherry plants and should not be interpreted as the incidence of disease specifically caused by *Phytopythium* spp. Although two plant samples were randomly collected from each of the three sectors, *Phy.* mycelia were isolated. From the plant showing decline symptoms, no *Phytophthora* spp. were recovered.

### 2.4. Evaluation of Pathogenicity in Cherry Plants

Pathogenicity assays showed that plants inoculated with all three *Phy.* isolates induced disease symptoms and root damage, whereas non-inoculated control plants remained asymptomatic. In the first experimental assay, non-inoculated control plants (T0) remained asymptomatic throughout the 90-day evaluation period, showed no root necrosis, and maintained greater shoot and root development than inoculated plants (Table 3 and Table S2; Figure 3). Although mean plant height, root length, and root fresh weight were generally lower in inoculated plants than in the control, treatment effects were not significant for plant height (F_3,16_ = 0.787, *p* = 0.518), root length (F_3,16_ = 2.428, *p* = 0.103), or root fresh weight (F_3,16_ = 1.956, *p* = 0.161). In contrast, inoculation treatment significantly affected shoot fresh weight (F_3,16_ = 8.056, *p* = 0.0017) (Supplementary Table S3). Tukey’s HSD test showed that plants inoculated with PUCV 2369 and PUCV 2372 had significantly lower shoot fresh weight than the non-inoculated control, whereas plants inoculated with PUCV 2368 showed an intermediate response and did not differ significantly from either the control or the other inoculated treatments.

Plants inoculated with PUCV 2368 (T1) exhibited loss of vigor, corresponding to a disease severity score of 1, together with 75% root necrosis. Plants inoculated with PUCV 2369 (T2) showed a disease severity score of 1 and 50% root necrosis, whereas plants inoculated with PUCV 2372 (T3) reached a disease severity score of 2 and 75% root necrosis. In contrast, control plants had a severity score of 0 and showed no root necrosis. Disease severity and root rot necrosis were analyzed using the Kruskal–Wallis test, followed by the Conover test for post hoc pairwise comparisons. In this analysis, all inoculated plants with *Phy* isolates differed significantly from the control plants, except for isolate PUCV 2369, which did not differ from the T0 treatment.

The independent experimental repeat showed a comparable pathogenic response (Supplementary Tables S4 and S5; Figure S1). Non-inoculated control plants remained asymptomatic and showed no root necrosis, whereas plants inoculated with PUCV 2368, PUCV 2369, and PUCV 2372 developed aboveground disease symptoms and root damage. In this experimental assay, all three inoculated treatments reached 70%, 75%, and 65% root necrosis and a disease severity score of 2, 2.2, and 2.4. Although absolute shoot and root growth measurements differed between experimental assays, the overall disease response was consistent: root necrosis and aboveground symptoms occurred in inoculated plants but were absent from non-inoculated controls. Taken together, the two independent experimental assays consistently reproduced disease development following inoculation with *Phy. helicoides*, supporting its pathogenic potential in sweet cherry plants.

Re-isolation of *Phy.* from inoculated plants was obtained in both independent pathogenicity assays (Supplementary Table S6; Figure S2). In the first assay, recovery frequencies were 100% (5/5 plants) for PUCV 2368, 60% (3/5) for PUCV 2369, and 100% (5/5) for PUCV 2372. In the second assay, recovery frequencies were 100% (5/5), 100% (5/5), and 80% (4/5) for PUCV 2368, PUCV 2369, and PUCV 2372, respectively. Re-isolated colonies showed morphological characteristics consistent with those of the corresponding original isolates. No *Phy.* was recovered from non-inoculated control plants (0/5) in either assay.

## 3. Discussion

This study provides evidence of an association between *Phy. helicoides* and root rot and decline symptoms in sweet cherry plants in Chile and demonstrates the pathogenicity of the evaluated isolates on *P. avium* ‘Lapins’ grafted onto MaxMa14. The combination of morphological characterization, molecular identification, phylogenetic analysis, and experimental inoculation provides complementary evidence for interpreting this association. Nevertheless, pathogenicity under experimental conditions should be distinguished from this pathogen’s contribution to the overall incidence of decline in the surveyed orchard.

Morphological analysis of PUCV 2368 and PUCV 2372 confirmed features typical of *Phy.*, such as papillate and non-papillate sporangia, internal proliferation, and discharge tube development. These traits align with the genus’s definition, which includes features shared with *Pythium* and *Phytophthora* [4]. The variation in dominant sporangial shapes between the two isolates suggests that identification should rely on a combination of characteristics rather than a single morphological trait. Sexual structures were not observed during the two-week period under the conditions used to induce sporangia. This indicates their absence in these conditions but does not rule out the isolates’ potential to produce sexual structures. Longer-term studies under different culture conditions are necessary to explore this aspect of their biology.

Molecular analyses confirmed that the isolates are *Phy. helicoides*, though results varied among markers for PUCV 2368. Sequences of ITS, β-tubulin, and cox1 from PUCV 2369 and PUCV 2372 showed the highest BLASTn identities with *Phy. helicoides*. Conversely, PUCV 2368’s ITS sequence matched a sequence labeled as *Phy. chamaehyphon*, while its β-tubulin and cox1 sequences aligned most closely with *Phy. helicoides*. The combined analysis positioned all three Chilean isolates within the *Phy. helicoides* clade. Using both nuclear and mitochondrial markers aligns with recommended practices for oomycete identification [22]. However, the combined placement does not clarify the conflicting ITS result. Therefore, interpret PUCV 2368’s identification based on the overall evidence, acknowledging that the source of the locus-specific discrepancy remains unresolved.

The identification of *Phy. helicoides* in symptomatic cherry roots supports the idea that this genus can impact woody hosts. Both *Phy. helicoides* and *Phy. vexans* are known as root and crown rot pathogens affecting various plants, making their detection important for nursery and orchard diagnosis [8]. Their significance in plant propagation is further demonstrated by cases of stem rot caused by *Phy. helicoides* in grafted ‘Shatangju’ mandarin seedlings in China [12]. Although the study involved a different host and disease type, it shows how this species can cause disease in young, grafted fruit plants.

The pathogenicity assays showed that all three isolates could cause root damage and aboveground symptoms in the tested sweet cherry–rootstock combination. However, growth responses varied among the variables assessed. In the first assay, plants inoculated with PUCV 2369 and PUCV 2372 had significantly lower shoot fresh weight than the non-inoculated control, while PUCV 2368 showed an intermediate effect. No significant treatment effects were observed for plant height, root length, or root fresh weight. These findings suggest that two isolates influence shoot fresh weight and promote disease development rather than causing a uniform reduction across all growth parameters. The lack of significant differences in some measures also indicates that visible root damage was not always associated with statistically detectable responses in every growth variable during the assessment period.

A relevant example within *Prunus* is the pathogenicity of *Phy. Vexans* on flowering cherry, *P. serrulata* ‘Kwanzan’. The study showed inoculated plants developed necrotic root lesions, the pathogen was re-isolated from symptomatic tissues, and control plants remained healthy [20]. These results offer a useful comparison with the root symptoms seen here, despite differences in host and pathogen species. They should be viewed as evidence of pathogenicity within the same host genus, not as proof that disease severity or growth responses are identical in flowering cherry and sweet cherry.

The two pathogenicity assays consistently showed that inoculated plants developed root necrosis and aboveground symptoms, while control plants did not exhibit root necrosis. The second assay offers supporting descriptive data to confirm the reproducibility of disease development, but its summary measures should not be used to infer treatment effects beyond the data’s support. Re-isolation of *Phy.* from inoculated roots strengthens the connection between inoculation and disease; however, since colonies were identified based on morphological comparison, this confirms the recovery of compatible colonies rather than conclusively verifying their identity via additional sequencing. Overall, the results indicate pathogenicity and suggest that further detailed comparisons among isolates are warranted.

Variation in disease responses has also been observed in other *Prunus* systems. Beluzán et al. [19] examined oomycetes on ‘Garnem’, ‘GF-677’, and ‘Rootpac-40’ hybrid rootstocks and noted differences in disease progression, with *Phy. helicoides* identified as one of the most virulent species tested [19]. In kiwifruit, the *Phy. helicoides* isolate evaluated by Prencipe et al. [13] caused more severe disease than the *Phy. chamaehyphon* isolate under their experimental conditions [13]. The pathogenicity of *Phy. chamaehyphon* on kiwifruit was also independently confirmed by [11]. These studies highlight that multiple members of the genus can induce root disease in perennial hosts, but their relative aggressiveness is not necessarily consistent across different hosts, isolates, and experimental setups. The current results do not support a consistent ranking of aggressiveness among the three Chilean isolates. Additional evidence of the broad potential host range of *Phy. helicoides* was provided by Christova [23], who reported susceptibility in 18 of 21 plant species evaluated using leaf and cutting inoculation assays. Although these findings demonstrate infection potential across diverse hosts, they should be distinguished from evidence of root disease in whole plants. Our study provides complementary evidence by demonstrating root necrosis and aboveground symptoms following substrate inoculation of sweet cherry plants grafted onto MaxMa14.

The orchard survey showed decline symptoms in 24.3–42.8% of plants across the three assessed sectors. These figures represent the observed decline, not necessarily caused by *Phy. helicoides*. The pathogen was recovered from symptomatic roots, and inoculation tests support its possible role in the root health issues. However, the limited sampling means we cannot determine what fraction of affected plants were infected with this pathogen. Additionally, not finding *Phytophthora* in the samples does not exclude its presence elsewhere in the orchard or the influence of other biological or environmental factors. Since the survey was conducted in only one commercial orchard, these incidence rates should not be generalized to all Chilean cherry production.

Differences among orchard sectors may result from variations in environmental conditions, plant material, management practices, or pathogen distribution, though we did not directly test these possibilities. Research on kiwifruit decline has highlighted the role of *Phy.* infection interacting with environmental factors such as waterlogging [13]. In cherry seedlings, longer flooding periods increased the severity of root rot caused by *Phytophthora cryptogea* and *Phytophthora megasperma* [24]. Although these findings concern a different oomycete genus, they support investigating soil saturation as a potential modifier of disease development in the sweet cherry–*Phy. helicoides* pathosystem. Using a zoospore suspension in the current assays provided a controlled way to expose roots to the pathogen, but it did not replicate natural inoculum levels or transmission pathways in the orchard. Future field research should combine pathogen detection with soil moisture, drainage, irrigation practices, and root health assessments to identify factors linked to disease development.

The occurrence of root necrosis despite variable aboveground responses highlights the value of including root examination in plant health assessments. In this study, root symptoms provided information that was not captured consistently by plant height or root biomass measurements. Reduced root function plausibly explains the lower shoot fresh weight observed in some inoculated treatments, but water uptake, nutrient acquisition, and physiological responses were not measured directly. Similarly, the experiment did not establish whether root necrosis preceded foliar symptoms or whether infection would reduce subsequent orchard productivity. These questions require assessments over time and longer-term evaluation after transplanting.

Several aspects of the study limit the generalizability of its findings. The research tested only three isolates and just one cultivar–rootstock combination, which restricts insights into pathogen diversity and susceptibility across different cherry rootstocks. Disease assessment was done visually by evaluators aware of the treatment groups, which, despite consistent criteria improving reliability, may still introduce observer bias. Future studies should incorporate blinded symptom scoring, quantitative root damage assessments, and a broader selection of isolates and plant genotypes. These approaches would better distinguish between the effects of different isolates and variations due to experimental conditions.

Potential sources of inoculum also require investigation. Barboza et al. [25] identified *Phy. chamaehyphon* and *Phy. oedochilum* in weeds from vegetable fields in Brazil [25]. This suggests that weeds near cherry orchards could serve as alternative hosts. However, the study did not focus on *Phy. helicoides* or cherry production, so the role of weeds as hosts or inoculum sources in this context remains unconfirmed. Conducting surveys of planting materials, irrigation water, soil, and nearby vegetation could help identify possible pathways for introduction and persistence. Integrated management of soilborne diseases can combine sanitation, resistant plant material, cultural practices, and biological or chemical control methods, with their suitability depending on the crop and pathogen involved [26]. These approaches provide a framework for future management studies on *Phy. helicoides* in sweet cherry. However, their effectiveness in this pathosystem requires experimental evaluation, as this study did not test any disease control measures.

The results confirm the pathogenic nature of the *Phy. helicoides* isolates tested on sweet cherry ‘Lapins’ grafted onto MaxMa14, indicating their possible role in causing root rot and decline in the surveyed Chilean orchard. Detecting these isolates highlights the importance of including *Phytopythium* in diagnostic tests for cherry root issues. Future research should explore the molecular differences seen in PUCV 2368, examine how the pathogen is distributed across orchards and nurseries, compare different cultivar–rootstock combinations, and assess management strategies suitable for local production conditions.

## 4. Materials and Methods

### 4.1. Pathogen Isolation and Morphological Characterization

Field root samples were processed by cutting approximately 10 mm fragments from the edge of the lesion observed in roots, rinsing them with sterile distilled water, and plating them on potato dextrose agar (PDA; Merck KGaA, Darmstadt, Germany) and corn meal agar (CMA; Merck KGaA, Darmstadt, Germany). The CMA medium was supplemented with pimaricin (10 mg L^−1^) (Merck KGaA, Darmstadt, Germany) and vancomycin (200 mg L^−1^) (Merck KGaA, Darmstadt, Germany), as a modification of the selective medium described by Tsao and Ocaña [27] to reduce bacterial contamination (hereafter MSP). Plates were incubated in the dark at 26 °C for 4 days. We subcultured emerging colonies onto MSP medium (corn meal agar and antibiotics) to promote stable mycelial growth of *Phytopythium* spp., following previously described protocols [28]. Cultures were maintained for 5–7 days at 24–25 °C in darkness.

We performed morphological characterization on two representative isolates (PUCV 2372 and PUCV 2368). We recovered the isolates and obtained mycelial discs (5 mm diameter) from the margin of actively growing colonies, transferred them to 90 mm Petri dishes with CMA medium in triplicate, and incubated the plates at 26 °C in darkness. We assessed colony growth after 48 h by measuring two perpendicular diameters of each colony and expressed radial growth as mm day^−1^. For microscopic observations, we generated sporangia according to established protocols for *Phytopythium* spp., based on Ghimire et al. [8], with minor adjustments.

Mycelial plugs (5 mm diameter) from actively growing cultures were transferred to liquid carrot juice medium, prepared by processing fresh carrots in a juice extractor and collecting the liquid fraction. We mixed 50 mL of the resulting carrot juice with 950 mL of distilled water and incubated it for 4 days at 25 °C in darkness. The mycelium was then washed 3–5 times with sterile distilled water to remove residual nutrients. To induce sporangia formation, mycelial fragments were exposed to a saline solution prepared with 2.36 g L^−1^ calcium nitrate (Ca(NO_3_)_2_), 0.50 g L^−1^ potassium nitrate (KNO_3_), and 1.00 g L^−1^ magnesium sulfate (MgSO_4_) in distilled water. The solution was supplemented with 1 mL L^−1^ of a stock solution containing 0.65 g EDTA, 0.375 g potassium hydroxide (KOH), and 1.225 g ferrous sulfate (FeSO_4_) (all chemical reagents were purchased from Merck KGaA, Darmstadt, Germany, and Sigma-Aldrich, St. Louis, MO, USA) in 50 mL of distilled water for 3 min, then transferred to sterile distilled water. Cultures were kept in the dark for 72 h until sporangia formation was confirmed.

Observations were made within two weeks of sporangial induction using a Nikon Eclipse-Ni microscope (Nikon Corporation, Tokyo, Japan) equipped with a Nikon DS-Fi2 digital camera. The length and width of at least 30 sporangia per isolate were measured.

### 4.2. Molecular Identification and Phylogenetic Analysis

Genomic DNA was extracted from mycelium of the *Phytopythium* isolates using the DNeasy Plant Mini Kit (QIAGEN GmbH, Hilden, Germany), following the manufacturer’s instructions. The extracted DNA was used to amplify three molecular markers: the internal transcribed spacer (ITS), β-tubulin, and mitochondrial cytochrome c oxidase subunit I (COX I) [29,30,31]. PCR amplifications were performed using SapphireAmp Fast PCR Master Mix (Takara Bio Inc., Kusatsu, Shiga, Japan) in a final reaction volume of 30 µL, containing 2 µL of genomic DNA and each primer at a final concentration of 0.42 µM.

The ITS region was amplified using primers ITS5/ITS4, following the cycling conditions recommended for SapphireAmp Fast PCR Master Mix, with an annealing temperature of 55 °C. We amplified the cox1 region using primers cox111-pyth-F1/cox111-pyth-R1, with an initial denaturation at 95 °C for 2 min, followed by 40 cycles of 94 °C for 30 s, 55 °C for 30 s, and 72 °C for 1 min, and a final extension at 72 °C for 7 min. The β-tubulin region was amplified using primers TUBUF2/TUBUR1 with an initial denaturation at 94 °C for 1 min, followed by 35 cycles of 98 °C for 5 s, 60 °C for 5 s, and 72 °C for 10 s.

PCR products were separated by electrophoresis on 1.5% agarose gels, and amplicons of the expected size were submitted to Macrogen Inc. (Seoul, Republic of Korea) for Sanger sequencing. Sequencing chromatograms were inspected and edited using Geneious software (v10.0.6; Biomatters Ltd., Auckland, New Zealand). The resulting sequences were compared with sequences available in the NCBI GenBank database using BLASTn for preliminary taxonomic identification.

For phylogenetic analyses, reference sequences of *Phy. helicoides* and phylogenetically related *Phytopythium* species were selected based on previous phylogenetic studies and retrieved from GenBank. ITS, β-tubulin, and *COX I* sequences were aligned separately using MUSCLE in MEGA version 12. For the multilocus analysis, sequences corresponding to the same reference isolates were concatenated in the order ITS–β-tubulin–*COX I*. The final concatenated dataset included 13 taxa and comprised 1549 aligned positions (525 for ITS, 814 for β-tubulin, and 210 for *COX I*). The best-fitting nucleotide substitution model was selected based on the Bayesian Information Criterion (BIC) [32]. Phylogenetic relationships were inferred using the Maximum Likelihood method under the Tamura–Nei model with gamma-distributed rate variation among sites (TN93+G), and branch support was evaluated using 1000 bootstrap replicates. All phylogenetic analyses were performed in MEGA version 12.

### 4.3. Field Disease Incidence

The incidence of decline symptoms was assessed during spring 2024 in three sectors of a single commercial sweet cherry orchard, designated Sector 1, Sector 2, and Sector 3. We visually evaluated 1260, 2285, and 2136 plants in these sectors, respectively.

Plants were classified as healthy, mildly affected, or moderately to severely affected based on a qualitative assessment of their overall condition, considering apparent reductions in plant height and vigor and the presence of foliar symptoms. Plants classified as mildly affected or moderately to severely affected were considered symptomatic for incidence calculations.

The incidence of decline symptoms was calculated separately for each sector by dividing the number of symptomatic plants by the total number of plants evaluated and multiplying by 100.

Root samples were collected from plants in each of the three sectors for pathogen isolation, as described in Section 4.1. Isolation was performed on a subset of the surveyed plants. Therefore, the incidence estimates represent the frequency of visually assessed decline symptoms rather than the incidence of disease specifically attributable to *Phy.*

All rootstocks in the surveyed orchard originated from the same nursery and were grafted in the orchard during winter.

### 4.4. Pathogenicity Assays

#### 4.4.1. Inoculum Production

The inoculum was prepared by obtaining sporangia as described above (Section 4.1). Zoospore release was induced by replacing the medium with cold sterile water (10 °C), followed by a temperature shift to 25 °C. The suspension was filtered through sterile gauze to remove mycelial fragments. Zoospore release and motility were verified microscopically before inoculation. Zoospore concentration was determined using a Neubauer chamber and adjusted to 1 × 10^4^ zoospores mL^−1^. Following zoospore release and concentration adjustment, the suspension was immediately applied to the root zone of the plants.

#### 4.4.2. Plant Material and Inoculation

Twelve-month-old *Prunus avium* cv. ‘Lapins’ plants grafted onto MaxMa14 rootstock were used for the pathogenicity assays. Plants were maintained in 3 L containers with sterilized peat–perlite substrate (3:1, *v*/*v*) and acclimated outdoors for 10 days before inoculation. The experiments were conducted during spring 2025 under outdoor greenhouse conditions at the La Palma Experimental Station (EELP), School of Agronomy, Pontificia Universidad Católica de Valparaíso (PUCV), Quillota, Chile. Plants were maintained under ambient spring temperature conditions; relative humidity was not monitored during the experiments.

The pathogenicity assay was performed twice as two independent experiments under the same experimental conditions. Each assay consisted of four treatments: a non-inoculated control (T0) and three inoculation treatments corresponding to *Phytopythium* isolates PUCV 2368 (T1), PUCV 2369 (T2), and PUCV 2372 (T3). Each treatment consisted of five biological replicates, with one plant per replicate, resulting in 20 plants per experiment. In both assays, the plants were disposed in a completely randomized design (CRD).

For inoculation, each plant received 300 mL of a freshly prepared zoospore suspension adjusted to 1 × 10^4^ zoospores mL^−1^, applied as a substrate drench to ensure distribution throughout the root zone. Control plants received the same volume (300 mL) of sterile distilled water (SDW). Following inoculation, the substrate was maintained under saturated conditions for 24 h to favor infection. Thereafter, plants were irrigated every three days and maintained under the same environmental conditions for 90 days post-inoculation [29].

#### 4.4.3. Disease Assay Evaluation

Disease severity was evaluated 90 days post-inoculation in both independent experiments. Parameters recorded included plant height (cm), fresh aerial biomass (including stem, branches and leaves) (kg), root length (cm), root fresh weight (complete harvested root system) and percentage of root necrosis. A severity scale adapted from Beluzán et al. [19] was applied as follows:

0 = asymptomatic plant;
1 = leaf yellowing and slight vigor reduction;
2 = wilting, partial defoliation, and dieback;
3 = dead plant.

The same evaluation criteria and experimental procedures were used for both separate experimental assays. Root necrosis was visually estimated as the percentage of the root system showing brown to dark necrotic lesions relative to the total root system.

#### 4.4.4. Pathogen Re-Isolation

To support confirmation of pathogenicity, we surface-disinfected four root fragments from each inoculated plant and plated them onto MSP medium. We processed each plant separately and considered it positive if we recovered *Phy.* from at least one of the four fragments. We compared re-isolated colonies with the corresponding original isolates based on colony morphology and microscopic features, using the diagnostic morphological characteristics of *Phy.* described by de Cock et al. [4]. Root fragments from non-inoculated control plants were processed using the same procedure. Re-isolation frequency was calculated for each treatment as the number of plants yielding *Phy.* divided by the total number of plants processed, multiplied by 100.

### 4.5. Statistical Analysis

Two independent pathogenicity experiments were conducted, each comprising four treatments with five biological replicates per treatment. Each plant constituted an experimental unit. Inferential statistical analyses were performed on the first experiment, for which complete plant-level data were available. We summarized results from the second experiment descriptively and present them in Supplementary Tables S4 and S5.

For the first experiment, the effects of inoculation treatment on plant height, shoot fresh weight, root length, and root fresh weight were evaluated using one-way analysis of variance (ANOVA). Residual normality and homogeneity of variances were assessed using the Shapiro–Wilk test and Levene’s test, respectively. When the overall treatment effect was significant, treatment means were compared using Tukey’s honestly significant difference (HSD) test. Continuous variables were summarized as means ± standard deviations (SD), with five plants per treatment.

Disease severity, assessed using an ordinal scale from 0 to 3, and visually estimated root necrosis, expressed as the percentage of affected root tissue, were evaluated at 90 days post-inoculation. For the first experiment, these variables were analyzed separately using the Kruskal–Wallis test, followed by Conover’s test for pairwise comparisons when the overall treatment effect was significant. Disease severity and root necrosis were summarized as medians, and statistical analyses used individual plant observations.

Statistical significance was assessed at *p* < 0.05. Re-isolation frequencies were summarized descriptively as the percentage of plants yielding *Phytopythium* within each treatment. No inferential comparisons were made between experiments or among treatments in the second experiment. Analyses were performed using R (R Core Team, Vienna, Austria).

## 5. Conclusions

This study provides evidence of the association of *Phy. helicoides* with root rot and decline symptoms in a commercial sweet cherry orchard in Chile. Morphological characterization and multilocus analyses supported the identification of the isolates, although the discordant ITS result for PUCV 2368 remains unresolved. Experimental inoculation demonstrated pathogenicity on *P. avium* ‘Lapins’ grafted onto MaxMa14, with root necrosis and aboveground symptoms observed in both assays. In the first assay, two isolates significantly reduced shoot fresh weight, whereas treatment effects on plant height, root length, and root fresh weight were not significant.

The incidence of visually assessed decline symptoms ranged from 24.3% to 42.8% across the three surveyed orchard sectors; these values do not represent disease incidence specifically attributable to *Phy. helicoides*. Together, the findings support its potential contribution to root health problems in the surveyed orchard and its inclusion in diagnostic investigations of sweet cherry decline. Further studies should assess its distribution, the susceptibility of other cultivar–rootstock combinations, and the environmental conditions associated with disease development.

## Figures and Tables

**Figure 1:**
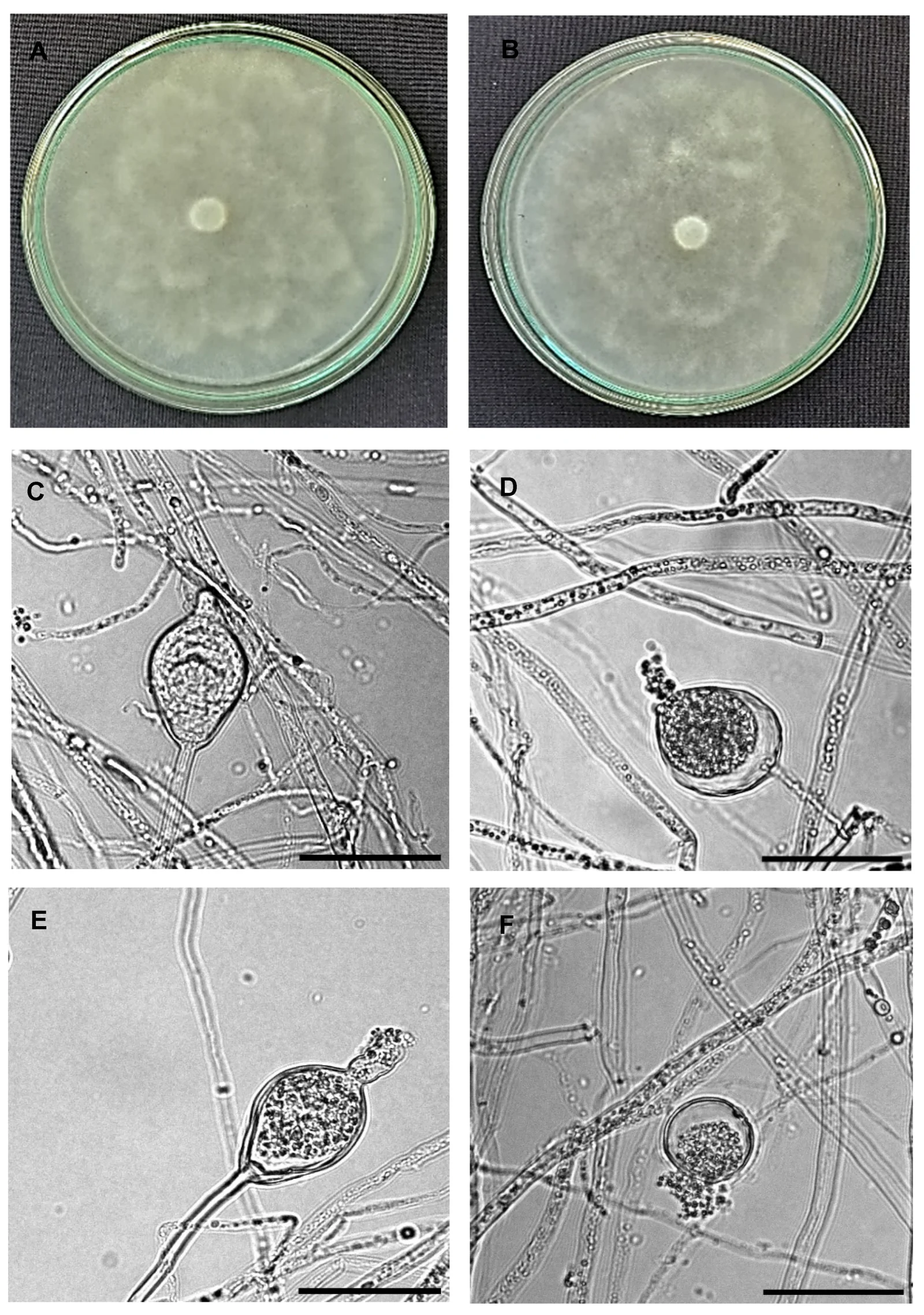
(**A**,**B**) Mycelial growth of isolates PUCV 2372 and PUCV 2368 on CMA plates incubated at 26 °C for 48 h. (**C**) Ellipsoid sporangium with papilla of isolate PUCV 2372; (**D**) Ovoid sporangium discharging zoospores of isolate PUCV 2368; (**E**) Discharge tube on ellipsoid sporangium of isolate PUCV 2372; (**F**) Globose sporangium of isolate PUCV 2368. Scale bars in (**C**–**F**) = 20 µm.

**Figure 2:**
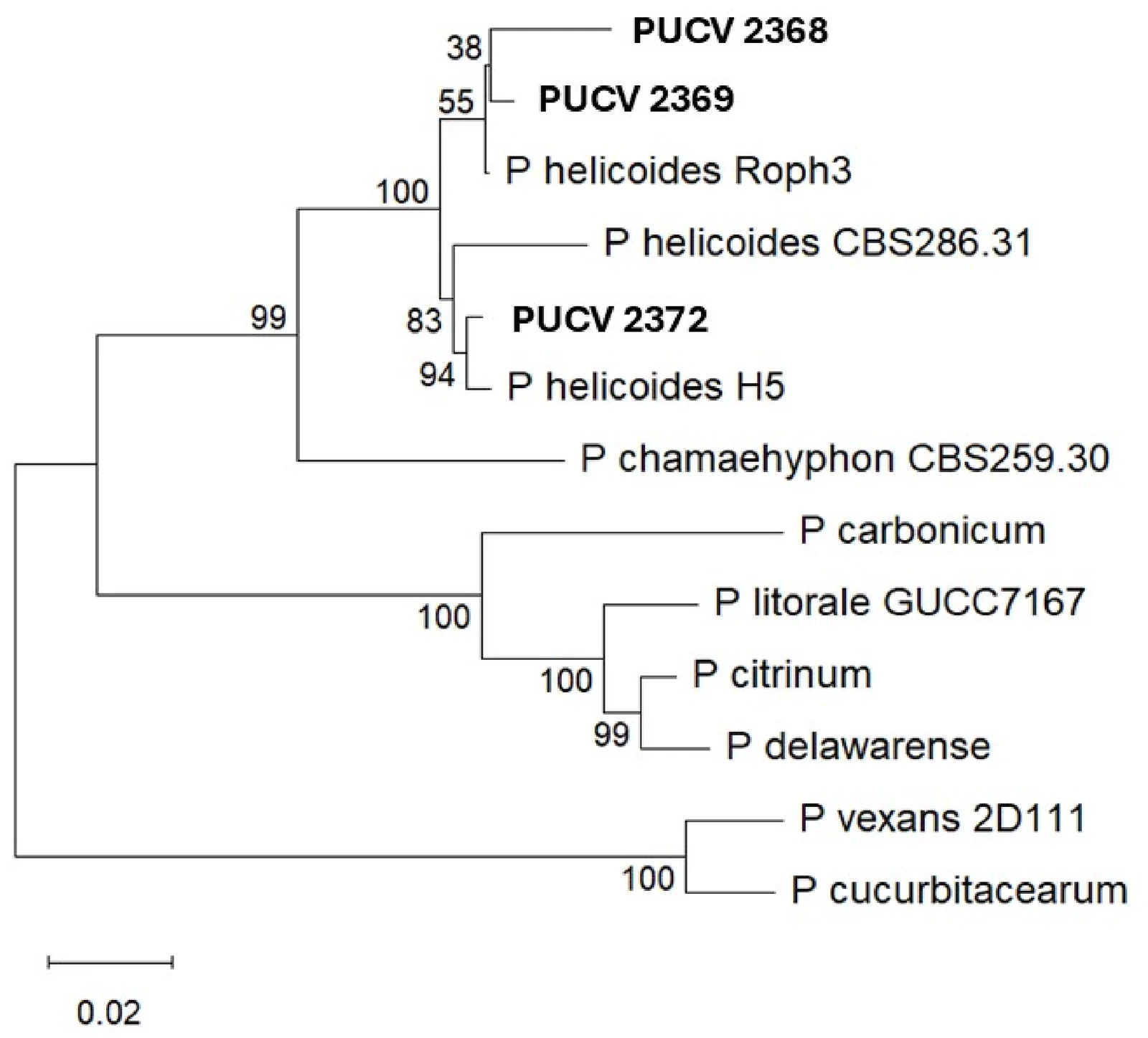
Phylogenetic trees comparing *Phytopythium* Chilean isolates with reference isolates, also analyzed using ITS, *cox1* and βt regions. *Phytopythium* reference species used in phylogenetic analysis are listed in Supplementary Material Table S1.

**Figure 3:**
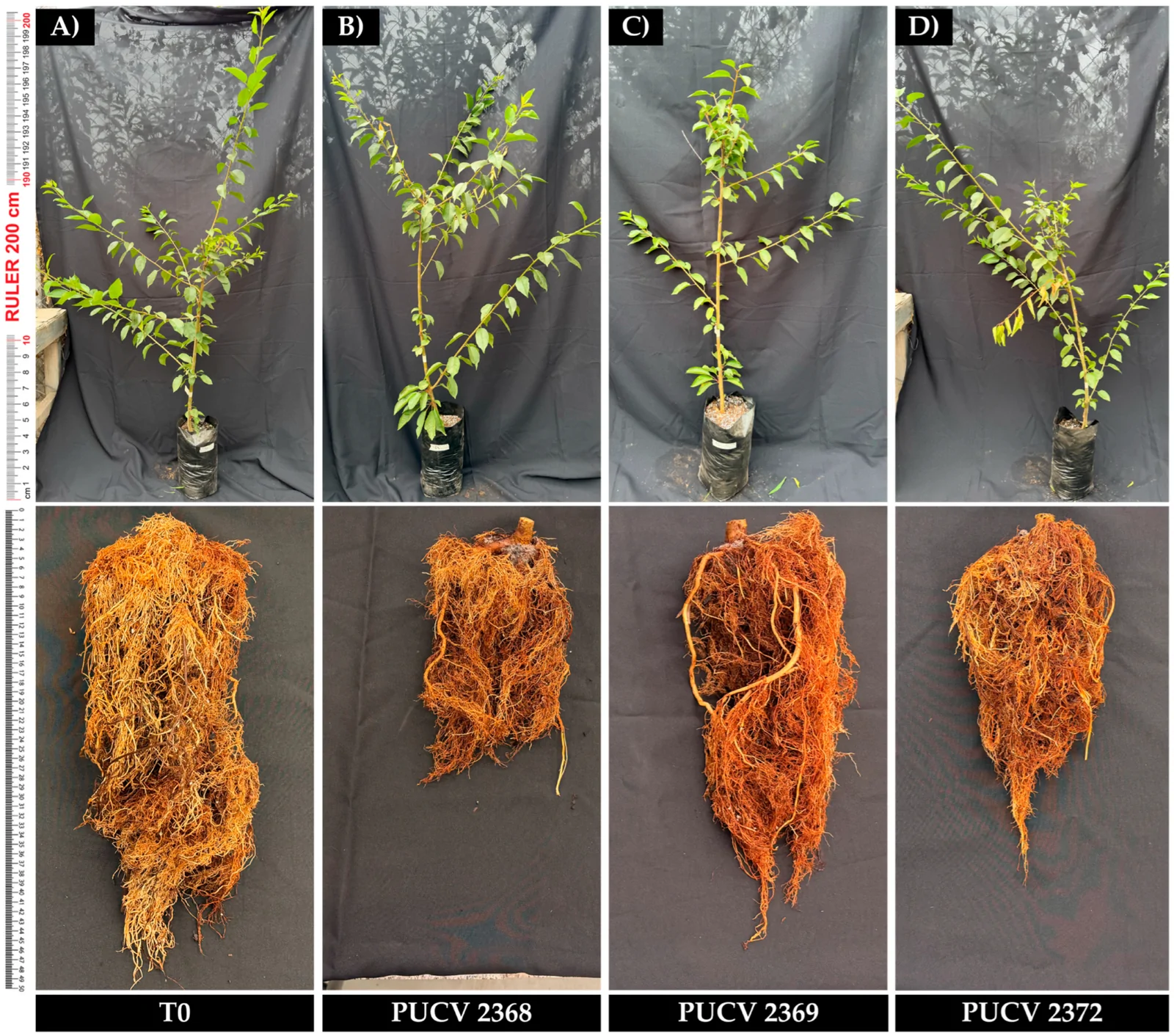
Representative ‘Lapins’ sweet cherry plants grafted onto MaxMa14 rootstock at 90 days post-inoculation in the first pathogenicity assay. One plant per treatment is shown: (**A**) non-inoculated control (T0); (**B**) plant inoculated with PUCV 2368 (T1), showing foliar chlorosis and reduced vigor (severity score 1); (**C**) plant inoculated with PUCV 2369 (T2), showing foliar chlorosis and reduced vigor (severity score 1); and (**D**) plant inoculated with PUCV 2372 (T3), showing wilting, partial defoliation, and dieback (severity score 2). The upper and lower panels show the aboveground portions and root systems, respectively.

**Table 1:** Closest matches obtained through BLASTn comparisons for ITS, β-tubulin, and COX I sequences of the three Chilean *Phytopythium* isolates.

Isolate	Closest BLASTn Match	GenBank Acc. Num	Length (bp)	Marker	Identity (%)
PUCV 2368	*Phy. chamaehyphon*	PP087944.1	623	ITS	100
	*Phy. helicoides*	AB948185.1	899	β-tubulin	99.65
	*Phy. helicoides*	LC798347.1	367	*COX I*	100
PUCV 2369	*Phy. helicoides*	PV849252.1	334	ITS	99.71
	*Phy. helicoides*	AB948185.1	905	β-tubulin	99.65
	*Phy. helicoides*	LC798347.1	230	*COX I*	99.13
PUCV 2372	*Phy. helicoides*	OR120473.1	381	ITS	99.21
	*Phy. helicoides*	AB948186.1	819	β-tubulin	99.13
	*Phy. helicoides*	PZ452032.1	215	*COX I*	99.53

**Table 2:** Incidence of visually assessed decline symptoms in ‘Lapins’ sweet cherry trees grafted onto Maxma14 rootstock across three sectors of a commercial orchard in the Valparaíso region, Chile.

Sector	Total Plants	N° of Plants with Decline Symptoms	Incidence (%)
Sector 1	1260	306	24.3
Sector 2	2285	978	42.8
Sector 3	2136	695	32.5

**Table 3:** Growth parameters and disease development in ‘Lapins’ sweet cherry plants grafted onto MaxMa14 rootstock following inoculation with *Phytopythium* isolates in the first pathogenicity assay, assessed at 90 days post-inoculation.

Treatments	Plant			Root		
	Height (cm)	Fresh Weight (kg)	Severity Scale	Length (cm)	Fresh Weight (kg)	Root Necrosis (%)
T0 (SDW)	149.6 ± 2.3 a	0.261 ± 0.027 a	0 a	40.3 ± 2.5 a	0.289 ± 0.074 a	0 a
T1 PUCV 2368	144.0 ± 8.4 a	0.207 ± 0.030 ab	1 b	37.0 ± 5.9 a	0.228 ± 0.074 a	75 b
T2 PUCV 2369	138.0 ± 15.7 a	0.189 ± 0.041 b	1 b	34.0 ± 3.4 a	0.212 ± 0.062 a	50 ab
T3 PUCV 2372	138.4 ± 20.9 a	0.167 ± 0.025 b	2 b	38.0 ± 1.9 a	0.201 ± 0.034 a	75 b

Values represent the mean ± SD of five biological replicates (*n* = 5). Different lowercase letters within each column indicate significant differences among treatments for continuous variables (plant height and fresh weight, root length and fresh weight) according to Tukey’s HSD test (*p* < 0.05). Disease severity and root necrosis are presented as descriptive data (median) and were analyzed using the Kruskal–Wallis test, followed by the Conover test for post hoc pairwise comparisons (*p* < 0.05). T0, non-inoculated control treated with sterile distilled water (SDW); T1, PUCV 2368; T2, PUCV 2369; T3, PUCV 2372.

## Data Availability

The data supporting the findings of this study are available within the article and its Supplementary Materials. Plant-level raw data from the pathogenicity assay are provided in Supplementary Tables S2 and S4.

## Supplementary Materials

The following supporting information can be downloaded at: https://www.mdpi.com/article/10.3390/plants15192926/s1, Table S1: Reference isolates of *Phytopythium* and GenBank accession numbers of the ITS, β-tubulin, and cox1 sequences used in the phylogenetic analysis; Table S2: Plant-level raw data for growth parameters in sweet cherry (*Prunus avium* ‘Lapins’) grafted onto MaxMa14 rootstock in the first pathogenicity assay; Table S3: One-way ANOVA and assumption tests for growth parameters in the first pathogenicity assay; Table S4: Plant-level raw data for growth parameters in sweet cherry (*Prunus avium* ‘Lapins’) grafted onto MaxMa14 rootstock in the second pathogenicity assay; Figure S1: Representative symptoms in sweet cherry plants in the second pathogenicity assay; Table S5: Effect of inoculation with *Phytopythium* isolates on plant growth and disease development in sweet cherry plants during the independent experimental repeat; Table S6: Re-isolation frequency of *Phytopythium* from sweet cherry roots in two independent pathogenicity assays; Figure S2: Representative cultures recovered from sweet cherry roots following inoculation in two independent pathogenicity assays.

## Abbreviations

The following abbreviations are used in this manuscript:

ANOVA	Analysis of Variance
BLASTn	Nucleotide sequences aligned to nucleotide database
CMA	Corn meal agar
COX I	Cytochrome c oxidase subunit I
DNA	Deoxyribonucleic acid
EDTA	Ethylenediaminetetraacetic acid
EELP	Estación Experimental La Palma
HSD	Honestly Significant Difference
ITS	Internal transcribed spacer
LSU	Large Subunit ribosomal RNA
MAFFT	Multiple Alignment using Fast Fourier Transform
MEGA	Molecular Evolutionary Genetics Analysis
MSP	Selective media for Phytophthora isolation
NCBI	National Center for Biotechnology Information
PCR	Polymerase Chain Reaction
PDA	Potato Dextrose agar
PUCV	Pontificia Universidad Católica de Valparaíso
SD	Standard deviation
SSU	Small subunit of ribosomal RNA
TAE	Tris-acetate-EDTA
TN93+G	Tamura-Nei nucleotide substitution model with gamma-distributed rate variation among sites
SDW	Sterile distilled water
