# Supplementary material for: Phytopythium helicoides Associated with Root Rot of Sweet Cherry in Chile: Identification, Pathogenicity, and Incidence of Decline Symptoms
Source: Plants (Basel). 2026 Sep 24;15(19):2926. doi: 10.3390/plants15192926 (PMC13666757; doi:10.3390/plants15192926)
Supplement: Supplementary file 1 [file plants-15-02926-s001.zip › plants-4513182-supplementary.pdf]

Supplementary material

Table S1. Reference isolates of *Phytopythium* and GenBank accession numbers of the ITS, β-tubulin, and cox1 sequences used in the phylogenetic analysis.

Species	Isolate	ITS accession no.	β-tubulin accession no.	cox1 accession no.
Phy. helicoides	CBS 286.31	AB725878	AB948187	AB690645
Phy. helicoides	H5	AB690611	AB948186	AB690633
Phy. helicoides	Roph3	AB690616	AB948185	AB690640
Phy. chamaehyphon	CBS 259.30	AB690609	AB948188	AB690644
Phy. litorale	GUCC 7167	AB690612	AB948182	AB690634
Phy. vexans	2D111	AB725880	AB948169	AB856784
Phy. carbonicum	–	AB725876	AB948183	AB690648
Phy. citrinum	–	AY197328	AB948180	AB690649
Phy. cucurbitacearum	–	AB725877	AB948189	AB690650
Phy. delawarense	–	AB725875	AB948181	AB690642

Phy. = *Phytopythium*; ITS = internal transcribed spacer region of nuclear ribosomal DNA; cox1 = mitochondrial cytochrome c oxidase subunit I gene. Accession numbers refer to reference sequences retrieved from GenBank.

Table S2. Plant-level raw data for growth parameters in sweet cherry (Prunus avium cv. 'Lapins') plants from the first pathogenicity assay.

Treatment	Isolate	Biological replicate	Plant height (cm)	Plant fresh weight (kg)	Root length (cm)	Root fresh weight (kg)
T0	Non-inoculated control	R1	150	0.2920	36.0	0.2152
T0	Non-inoculated control	R2	152	0.2493	42.0	0.3568
T0	Non-inoculated control	R3	146	0.2797	42.0	0.2157
T0	Non-inoculated control	R4	149	0.2600	40.5	0.2900
T0	Non-inoculated control	R5	151	0.2231	41.0	0.3697
T1	PUCV 2368	R1	137	0.1833	40.0	0.2138
T1	PUCV 2368	R2	158	0.2527	36.0	0.2015
T1	PUCV 2368	R3	143	0.1777	28.0	0.1465
T1	PUCV 2368	R4	144	0.2000	37.0	0.2300
T1	PUCV 2368	R5	138	0.2193	44.0	0.3474
T2	PUCV 2369	R1	160	0.2531	38.0	0.2985
T2	PUCV 2369	R2	143	0.1607	33.0	0.1462
T2	PUCV 2369	R3	117	0.1451	29.0	0.1609
T2	PUCV 2369	R4	138	0.1900	34.0	0.2100
T2	PUCV 2369	R5	132	0.1957	36.0	0.2448
T3	PUCV 2372	R1	150	0.1700	38.0	0.1613
T3	PUCV 2372	R2	159	0.1781	37.0	0.1735
T3	PUCV 2372	R3	104	0.1257	41.0	0.2298
T3	PUCV 2372	R4	138	0.1700	38.0	0.2000
T3	PUCV 2372	R5	141	0.1916	36.0	0.2397

Table S3. One-way ANOVA and assumption tests for growth parameters in the first pathogenicity assay.

Variable	Df	F	ANOVA *p*	Shapiro-Wilk *p*	Levene *p*
Plant height	3,16	0.787	0.518	0.043	0.236
Fresh weight	3,16	8.056	0.0017	0.719	0.756
Root length	3,16	2.428	0.103	0.299	0.319
Root fresh weight	3,16	1.956	0.161	0.464	0.600

Table S4. Plant-level raw data for growth parameters in sweet cherry (*Prunus avium* cv. 'Lapins') plants from the second pathogenicity assay.

Treatment	Isolate	Replicate	Plant height (cm)	Fresh weight (kg)	Root length (cm)	Root fresh weight (kg)
T0	Non-inoculated control	R1	166.11	0.1000	40.02	0.2800
T0	Non-inoculated control	R2	168.36	0.1000	41.83	0.2800
T0	Non-inoculated control	R3	170.61	0.7000	43.65	0.3200
T0	Non-inoculated control	R4	172.86	1.3000	45.47	0.3600
T0	Non-inoculated control	R5	175.11	1.3000	47.28	0.3600
T1	PUCV 2368	R1	154.79	0.4900	28.04	0.1900
T1	PUCV 2368	R2	159.90	0.4900	30.52	0.1900
T1	PUCV 2368	R3	165.00	0.5000	33.00	0.2600
T1	PUCV 2368	R4	170.10	0.5100	35.48	0.3300
T1	PUCV 2368	R5	175.21	0.5100	37.96	0.3300
T2	PUCV 2369	R1	162.81	0.2675	25.09	0.2000
T2	PUCV 2369	R2	165.65	0.2675	27.55	0.2000
T2	PUCV 2369	R3	168.50	0.2675	30.00	0.2200
T2	PUCV 2369	R4	171.35	0.2675	32.45	0.2400
T2	PUCV 2369	R5	174.19	2.2800	34.91	0.2400
T3	PUCV 2372	R1	152.13	0.4600	29.43	0.2300
T3	PUCV 2372	R2	156.36	0.4600	31.51	0.2300
T3	PUCV 2372	R3	160.60	0.4800	33.60	0.2900
T3	PUCV 2372	R4	164.84	0.5000	35.69	0.3500
T3	PUCV 2372	R5	169.07	0.5000	37.77	0.3500

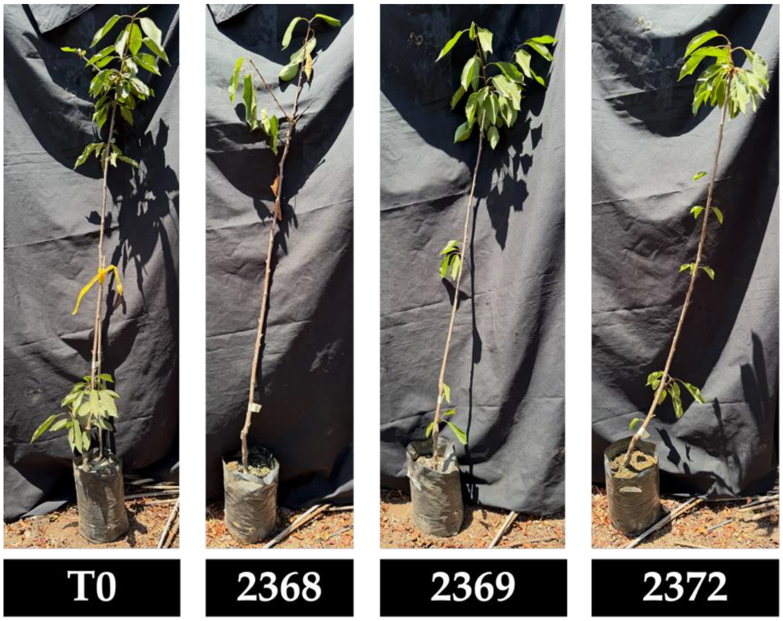

Figure S1. Symptoms of disease in sweet cherry (*Prunus avium* cv. Lapins) plants observed during a separate pathogenicity test with *Phytopythium* species. The image shows representative plants 90 days post-inoculation. From left to right: T0, non-inoculated control; PUCV 2368 ; PUCV 2369; and PUCV 2372. Inoculated plants exhibited varying levels of reduced vigor, foliar wilting, and leaf loss compared to the non-inoculated control.

Table S5. Effect of inoculation with ***Phytopythium*** isolates on plant growth and disease development in sweet cherry plants during the independent experimental repeat.

Treatments	Plant			Root		
	Height (cm)	Fresh weight (kg)	Severity scale	Lenght (cm)	Fresh weight (kg)	% necrosis
T0	170.61 ± 3.56 a	0.70 ± 0.6 a	0 a	43.65 ± 2.87 a	0.32 ± 0.04 a	0 a
T1	165.0 ± 8.07 a	0.50 ± 0.01 a	2 b	33.0 ± 3.92 b	0.26 ± 0.07 a	70 b
T2	168.5 ± 3.67 a	0.67 ± 0.9 a	2,2 b	30.0 ± 3.88 b	0.22 ± 0.02 a	75 b
T3	160.6 ± 6.70 a	0.48 ± 0.02 a	2,4 b	33.6 ± 3.3 b	0.29 ± 0.06 a	65 b

Values represent mean ± SD of five biological replicates (n = 5). T0, non-inoculated control; T1, PUCV 2368; T2, PUCV 2369; T3, PUCV 2372.

Table S6. Recovery frequency of *Phytopythium* spp. from sweet cherry roots following experimental inoculation in two independent pathogenicity assays.

Assay	Treatment	Inoculated isolate	Positive plants / total plants	Recovery (%)
Assay 1	T0	Non-inoculated control	0/5	0
Assay 1	T1	PUCV 2368	5/5	100
Assay 1	T2	PUCV 2369	3/5	60
Assay 1	T3	PUCV 2372	5/5	100
Assay 2	T0	Non-inoculated control	0/5	0
Assay 2	T1	PUCV 2368	5/5	100
Assay 2	T2	PUCV 2369	5/5	100
Assay 2	T3	PUCV 2372	4/5	80

Note: Four root fragments were plated from each plant. A plant was considered positive when *Phytopythium* was recovered from at least one of the four root fragments. Re-isolated colonies were identified by morphological comparison with the corresponding original isolates.

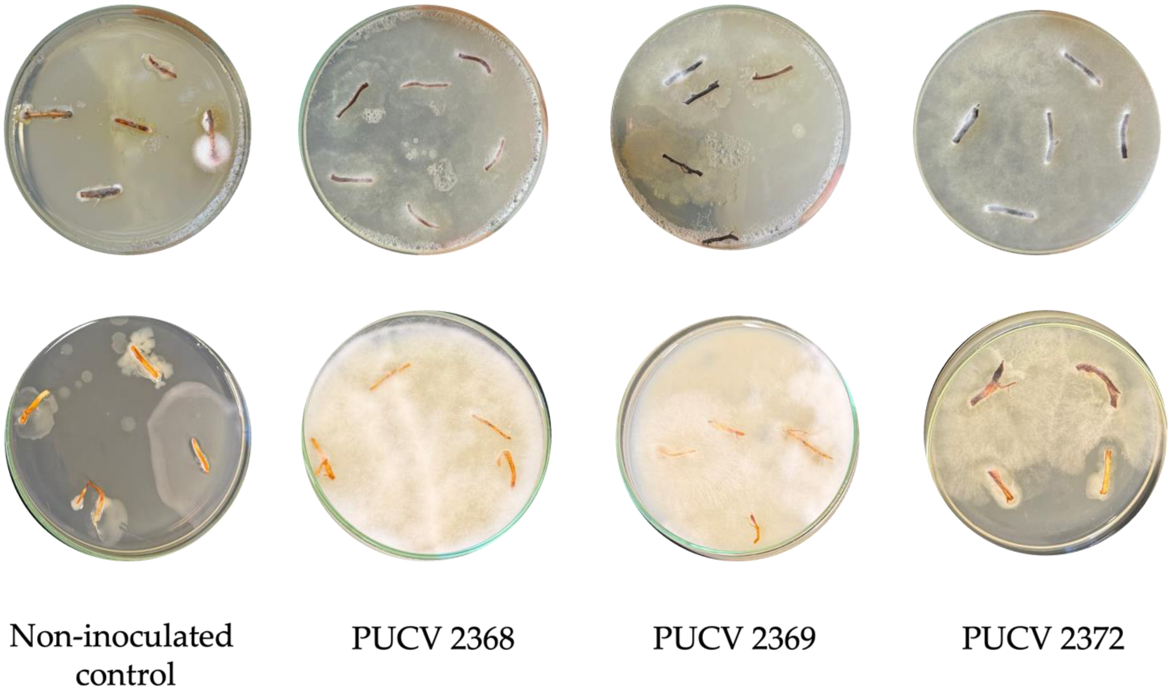

Figure S2. Representative re-isolation of *Phytopythium* spp. from sweet cherry (*Prunus avium* cv. 'Lapins') roots following experimental inoculation in two independent pathogenicity assays. Root fragments were collected at the end of each 90-day assay and plated onto selective medium. Representative plates are shown for the non-inoculated control (T0) and plants inoculated with PUCV 2368 (T1), PUCV 2369 (T2), and PUCV 2372 (T3). We identified re-isolated colonies by morphological comparison with the corresponding original isolates.
